# Effect of growth hormone treatment on energy expenditure and its relation to first-year growth response in children

**DOI:** 10.1007/s00421-018-4033-6

**Published:** 2018-11-26

**Authors:** Saartje Straetemans, D. A. Schott, Guy Plasqui, Hilde Dotremont, Angèle J. G. M. Gerver-Jansen, An Verrijken, Klaas Westerterp, Luc J. I. Zimmermann, Willem-Jan M. Gerver

**Affiliations:** 1 Department of Paediatric Endocrinology, Maastricht University Medical Center, P. Debyelaan 25, 6229 HX Maastricht, The Netherlands; 2 NUTRIM School of Nutrition and Translational Research in Metabolism, Maastricht University, Maastricht, The Netherlands; 3 Department of Paediatrics, Zuyderland Medical Center, Henri Dunantstraat 5, 6419 PC Heerlen, The Netherlands; 4 Department of Human Biology, Maastricht University, Minderbroedersberg 4-6, 6211 LK Maastricht, The Netherlands; 5 Department of Paediatric Endocrinology, Antwerp University Hospital, Wilrijkstraat 10, 2650 Edegem, Belgium; 6 Department of Endocrinology, Diabetology and Metabolic Diseases, Antwerp University Hospital, Wilrijkstraat 10, 2650 Edegem, Belgium; 7 Laboratory of Experimental Medicine and Paediatrics, Faculty of Medicine and Health Sciences, University of Antwerp, Universiteitsplein 1, 2610 Antwerp, Belgium; 8 Paediatrics Department, Maastricht University Medical Center, P. Debyelaan 25, 6229 HX Maastricht, The Netherlands

**Keywords:** Energy expenditure, Body composition, Metabolism, Growth hormone treatment, Children, First-year growth response

## Abstract

**Purpose:**

The effects of growth hormone (GH) treatment on linear growth and body composition have been studied extensively. Little is known about the GH effect on energy expenditure (EE). The aim of this study was to investigate the effects of GH treatment on EE in children, and to study whether the changes in EE can predict the height gain after 1 year.

**Methods:**

Total EE (TEE), basal metabolic rate (BMR), and physical activity level (PAL) measurements before and after 6 weeks of GH treatment were performed in 18 prepubertal children (5 girls, 13 boys) born small for gestational age (*n* = 14) or with growth hormone deficiency (*n* = 4) who were eligible for GH treatment. TEE was measured with the doubly labelled water method, BMR was measured with an open-circuit ventilated hood system, PAL was assessed using an accelerometer for movement registration and calculated (PAL = TEE/BMR), activity related EE (AEE) was calculated [AEE = (0.9 × TEE) − BMR]. Height measurements at start and after 1 year of GH treatment were analysed. This is a 1-year longitudinal intervention study, without a control group for comparison.

**Results:**

BMR and TEE increased significantly (resp. 5% and 7%). Physical activity (counts/day), PAL, and AEE did not change. 11 out of 13 patients (85%) with an increased TEE after 6 weeks of GH treatment had a good first-year growth response (∆height SDS > 0.5).

**Conclusions:**

GH treatment showed a positive effect on EE in prepubertal children after 6 weeks. No effect on physical activity was observed. The increase in TEE appeared to be valuable for the prediction of good first-year growth responders to GH treatment.

## Introduction

Already for many years, short children with growth hormone (GH) deficiency (GDH) and/or born small for gestational age (SGA) have been treated with recombinant human GH to promote their linear growth. Beside its growth-promoting effect, GH has many specific metabolic effects as well, including (1) increased mobilization of fatty acids from adipose tissue and increased use of fatty acids for energy, (2) increased rate of protein synthesis in most cells of the body, and (3) decreased rate of glucose utilization throughout the body. The effect of these changes in metabolism is reflected in a decrease in fat mass and an increase in fat free mass, as shown in several studies (Vaisman et al. 1994, 1992; Gregory et al. 1991, 1993; Ernst et al. 2012; Khadilkar et al. 2014; Walker et al. 1990; Boot et al. 1997; Hassan et al. 1996).

Beside a change in body composition, it is reasonable to assume that also energy expenditure will be influenced by GH treatment. Total daily energy expenditure (TEE) can be divided into 3 components: (1) basal metabolic rate (BMR), the amount of energy required to maintain all vital body functions at rest with no additional activity; (2) diet-induced thermogenesis (10%) (Westerterp 2004); (3) activity related energy expenditure (AEE). However, very little research has been done on changes in energy expenditure caused by GH treatment in children. Vaisman et al. (1994) showed an increase in BMR after 2 months of GH treatment in 10 prepubertal boys. Gregory et al. (1991) were the first and up to now the only who studied GH effects on BMR as well as TEE in 15 children. They demonstrated a significant increase in BMR and TEE after 6 weeks of GH treatment.

It has been shown that changes in body composition can predict the growth response after the first year of GH treatment. Hoos et al. (2003b) showed a strong relationship between the GH induced first-year growth response and the increase in total body water (TBW)/height^2^ after 6 weeks in 28 prepubertal children suspected of being GH deficient. Eighty percent of the children with a good growth response (increase in height SDS > 0.7) had a change in TBW/height^2^ exceeding the 2 SD reference line of the control group. Additionally, Ernst et al. (2012) showed that the change in TBW after 6 weeks of GH treatment correctly predicted the growth response after the first year in 75% of GHD patients (*n* = 88). For children born SGA (*n* = 99), a change in TBW of > 0.7 L/m^2^ was strongly predictive for a good growth response, but the negative predictive value was low (30%). Gregory et al. (1993) showed in 15 children that not only body composition but also 6 weeks changes in energy expenditure were correlated with height velocity increases at 6 months of GH treatment.

The first aim of this study is to investigate the effects of GH on energy expenditure (BMR, TEE and AEE) and body composition in prepubertal children. Our hypothesis is that the changes in body composition are related to changes in energy expenditure after 6 weeks of GH treatment in children. The second aim of this study is to investigate the relation of the GH induced changes in energy expenditure and the height gain after 1 year. We hypothesize that the increased energy expenditure after 6 weeks of GH treatment can predict the height gain after the first year of GH treatment.

## Subjects and methods

### Study design

This was a prospective study, approved by the Medical Ethical Research Committee of the University of Maastricht and the Antwerp University Hospital. Informed consent is secured prior to entry in the study. This is a 1-year longitudinal intervention study, without a control group for comparison.

### Patients

Children visiting the outpatient clinic at the Maastricht University Medical Center and the Antwerp University Hospital were screened by paediatric endocrinologists for participation in the study. Children aged ≥ 4 years with GHD and/or born SGA without catch-up growth and who were scheduled for treatment with recombinant human GH on a daily regimen for at least 1 year, were eligible for participation. The diagnosis of GHD was made by the treating paediatric endocrinologist according to international guidelines, including a height velocity (HV) below the 25th percentile, a low IGF-I concentration, a delayed bone age and a peak GH concentration below 20 mIU/L in 2 GH provocation tests (glucagon, arginine and/or insulin test). Children born SGA without catch-up growth had to meet the following inclusion criteria: (1) a birth length and/or weight < − 2.0 standard deviation (SD); (2) height at start of GH treatment < − 2.5 SD; (3) height at start of GH treatment ≥ 1.0 SD below target height SD score (SDS). Exclusion criteria were: (1) chronological or bone age greater than 8 years for girls and 9 years for boys; (2) puberty during first year of GH treatment (girls Tanner breast stage ≥ 2, boys testicular volume ≥ 4 mL); (3) syndromes or diseases that influence growth other than GDH or SGA; (4) concomitant treatment with glucocorticosteroids (> 12 mg/m^2^/day hydrocortisone equivalent) in preceding year or during first-year GH treatment; (5) previous or current treatment with other growth stimulating medications (e.g., sex steroids, oxandrolone, letrozole); (6) other pituitary hormone deficiencies present at start or during first-year GH therapy. If a patient met the inclusion and exclusion criteria, the study was explained to the patients/parents and they were asked whether they were interested in taking part in the study. During the enrollment period only 2 patients did not participate because the parents did not have time for the ventilated hood measurements. Patients were treated with subcutaneous injections of recombinant human GH at a dose of 35 µg/kg day for children born SGA, and 25 µg/kg day for children with GHD.

### Methods

Auxological parameters (height and weight) were measured at start, after 6 weeks, and after 1 year of GH treatment. A stadiometer accurate to 0.1 cm was used for all height measurements. Weight was measured using an electric scale accurate to 0.1 kg with the patient only wearing underwear.

Birth weight for gestational age was transformed into SDS, based on the standards of Niklasson et al. (1991). The midparental height (MPH) (cm) was calculated as (father’s height + mother’s height + 13)/2 for boys and (father’s height + mother’s height − 13)/2 for girls (Cole 1996). Height, weight, body mass index (BMI), MPH and HV were converted to SDS [(patient parameter − mean of the reference population)/SD of the reference population] using the Belgian reference data by Roelants et al. (2009). An increase in height SDS < 0.5 was defined as a poor first-year growth response (Bang et al. 2012).

#### Total energy expenditure (TEE) and total body water (TBW)

The doubly labelled water (DLW) method, according to the Maastricht protocol was used for the measurement of body composition and TEE before and 6 weeks after start of GH treatment. This isotope technique is validated by comparing measurements with results from alternative techniques, and by analysis of the reproducibility within subjects and within observations (Westerterp et al. 1995; Westerterp 1999a). It is the golden standard method. A baseline urine sample was collected. Then, a weighed isotope dose of DLW, a mixture of 10% ^18^O and 5% ^2^H in water, was orally administered. Children drank the water straight from the bottle (~ 70 mL) after which the bottle was partly refilled with tap water which was also consumed, to be sure the complete dose of DLW was ingested. The children drank the water in the evening before they went to bed. The next morning, when equilibration of the isotope with the body water had occurred, a urine sample was collected from the second voiding. The DLW and urine samples were stored in air-tight, screw-capped glass containers. TEE was measured over a 2-week period, thus collection of urine samples were repeated at day 8 and 14. Sample analysis requires a sophisticated laboratory with an isotope-ratio mass spectrometer and a sample preparation system. The department of human biology at the Maastricht University in Maastricht, The Netherlands fulfils these requirements and analysed all samples. The samples were analysed in duplicate with an isotope-ratio mass spectrometer (Optima, VG Isogas, Cheshire, UK).

CO_2_ production was calculated from the difference in disappearance rates of both isotopes, as calculated from the slope of the elimination curves.

Oxygen consumption was then calculated from measured CO_2_ production by assuming an average RQ of 0.85, representative of a normal mixed diet (Black et al. 1986). Energy expenditure was then calculated using Weir’s formula (1949).

Fat free body mass was calculated from TBW using the age-specific fat-free mass hydration constants for children by Lohman (1989).

#### Basal metabolic rate (BMR) and physical activity level (PAL)

Basal metabolic rate was measured with an open-circuit, ventilated hood system before and 6 weeks after GH treatment (Adriaens et al. 2003). It was measured in the morning after an overnight fast to avoid diet-induced thermogenesis being included in the measurement. The subjects were asked to lie in supine position for 30 min. Oxygen consumption and carbon dioxide production were calculated using the flow through the hood and the oxygen and carbon dioxide concentrations in the incoming and outcoming air using the Omnical system at the Maastricht University, The Netherlands and the CareFusion, Respiratory Diagnostics, SensorMedics Vmax Encore at the Antwerp University Hospital, Belgium. The Omnical was calibrated daily and validated weekly using methanol burns. The CareFusion was calibrated before every measurement. BMR was calculated from oxygen consumption and carbon dioxide production using Weir’s equation (Weir 1949). Once TEE and BMR were known, PAL was calculated as TEE/BMR (Human energy requirements. Scientific background papers from the Joint FAO/WHO/UNU Expert Consultation. October 17–24 2001. Rome, Italy 2005).

Physical activity was also assessed using a Direct Life tri-axial accelerometer for movement registration (Tracmor^®^) (Philips New Wellness Solutions; http://www.directlife.philips.com) (Bonomi et al. 2010; Hoos et al. 2003a). The Tracmor has been developed at the department of Human Biology at the University of Maastricht. It has proved to be an objective and reliable tool for assessing activity levels in free-living subjects (Westerterp 1999b). In contrast to other accelerometers, Tracmor was miniaturized to a small (3.2 × 3.2 × 0.5 cm) and light (13 g) device, which is important for the subject’s comfort (Westerterp 2001). The Tracmor^®^ was placed at the lower back of the child using an elastic belt. The child was instructed to wear the accelerometer during daytime. At the end of the monitoring period the Tracmor^®^ was connected to a personal computer and the recorded data were downloaded using dedicated software. Tracmor^®^ output was expressed as activity counts/minute. The Tracmor^®^ activity counts/minute were summed over the entire monitoring period and divided by the number of monitoring days to determine the average Tracmor^®^ counts per day (Cnts/d).

Activity related energy expenditure (AEE) was calculated as (0.9 × TEE) − BMR, assuming a diet-induced thermogenesis (DIT) of 10% (Westerterp 2004).

### Statistical analysis

The variables are reported as the mean ± SD. A Shapiro–Wilk test was used to test for the normal distribution. Differences between groups were tested with a *t* test when the distribution of data was normal, and with a Mann–Whitney *U* test otherwise. Significance is considered at the 5% level (*p* < 0.05). IBM SPSS statistics^®^ (version 21) was used for all statistical analyses.

## Results

Eighteen subjects were enrolled. The ventilated hood method was used in all subjects for BMR measurements. The doubly labelled water method was used in all subjects for TEE and body composition measurements. Unfortunately, due to technical problems, the ventilated hood results of 6 subjects were unusable. Therefore, we have BMR results of 12 subjects and TEE and body composition results of 18 subjects.

### Background and baseline characteristics

The background and baseline auxological characteristics of 18 children (5 girls, 13 boys) born SGA (*n* = 14) or with idiopathic GHD (*n* = 4) who started GH treatment are listed in Table 1.

**Table 1:** Subject characteristics

	*n*	Mean	SD
Gestational age, weeks	17	36.5	3.2
Birth weight, SDS	17	− 2.02	1.22
Birth length, SDS	15	− 2.18	1.30
Father height, SDS	18	− 0.9	1.20
Mother height, SDS	18	− 0.86	1.06
MPH, SDS	18	− 0.92	0.82
At start GH treatment			
Age, years	18	6.4	1.5
Height, SDS	18	− 2.92	0.85
Height SDS minus MPH SDS	18	− 2.00	0.81
Weight, SDS	18	− 2.51	1.07
BMI, SDS	18	− 0.52	0.89
Pretreatment HV, SDS^a^	4	− 0.9	0.7
Bone age delay, years^a^	4	1.7	1.1
IGF-1, SDS^a^	4	− 2.1	0.3
Maximum GH peak, mIU/L^a^	4	16.6	0.3
GH dose, µg/kg day	18	35.5	4.4
After first-year GH treatment			
Height, SDS	18	− 2.15	0.81
∆ height, SDS	18	0.73	0.37

*MPH* midparental height, *GH* growth hormone, *BMI* body mass index, *HV* height velocity, *IGF-1* insulin like growth factor 1, *∆ height SDS* increase in height SDS during first-year GH treatment

^a^Data only of GHD patients

The children started GH treatment at a mean age of 6.4 years and a mean height of − 2.92 SD. They were short for their parents (height SDS minus MPH SDS − 2.00). There was no significant difference between girls and boys.

### Body composition

**Fig. 1:**
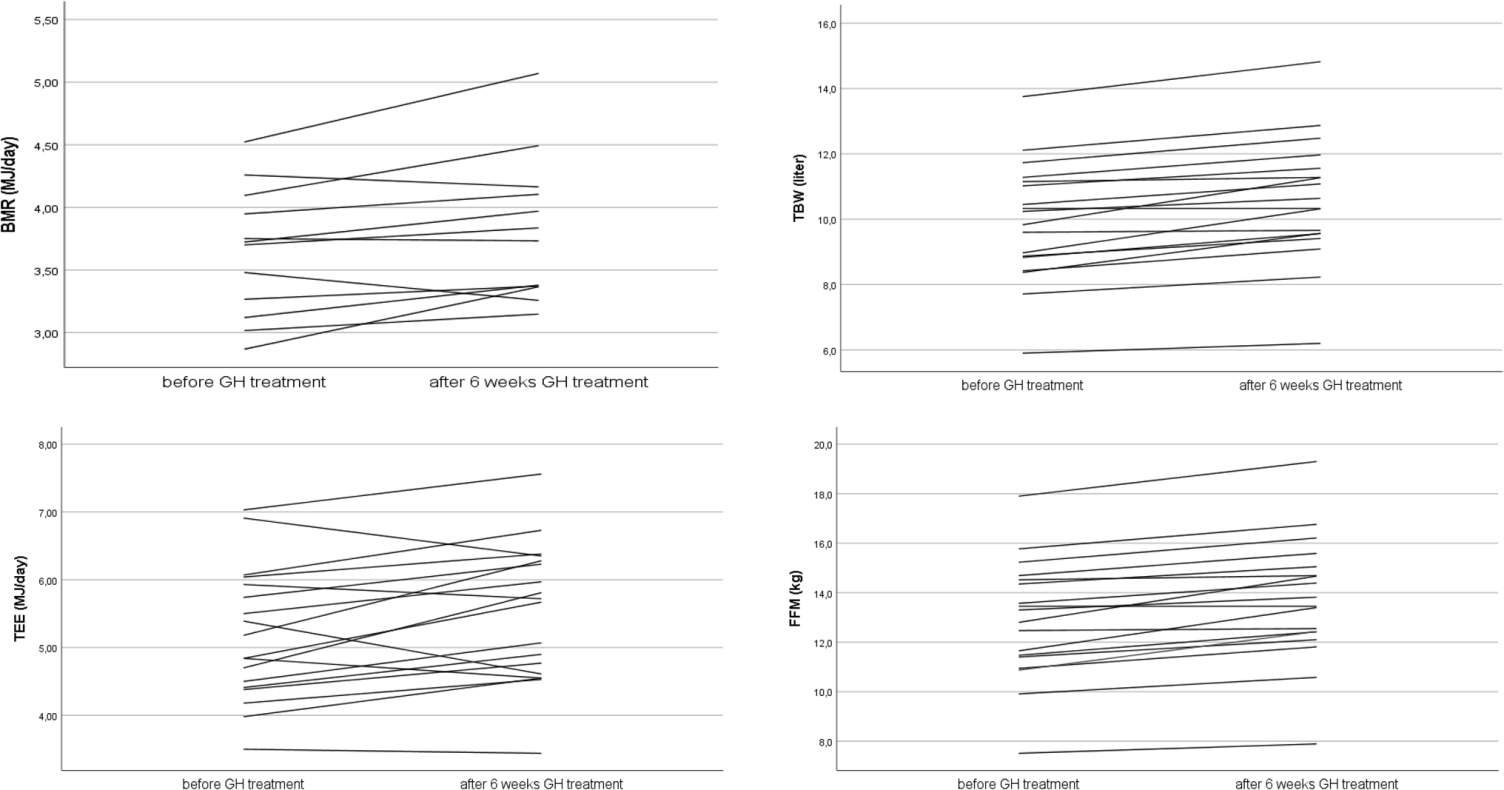
Changes in total body water (TBW), fat free mass (FFM), basal metabolic rate (BMR), and total energy expenditure (TEE) after 6 weeks of growth hormone (GH) treatment for each individual subject

The body composition of 18 children before and after 6 weeks of GH treatment is given in Table 2. There was a significant increase in body weight after 6 weeks of GH treatment. The increase in TBW (0.7 ± 0.4 L; 95% CI 0.45–0.86; *p* < 0.001) and FFM (0.9 ± 0.5 kg; 95% CI 0.6–1.1; *p* < 0.001) after 6 weeks of GH treatment was significant. There was no significant difference between girls and boys. Figure 1 illustrates the changes in TBW and FFM for each individual subject.

**Table 2:** Body composition before and after 6 weeks of GH treatment

	*n*	Before GH treatment		After 6 weeks GH treatment		Delta			*p* value
		Mean	SD	Mean	SD	Mean	SD	95% CI	
Body weight, kg	18	16.7	3.4	17.1	3.4	0.5	0.6	0.2 to 0.8	< 0.05
BMI, SDS	18	− 0.52	0.89	− 0.43	0.81	0.08	0.36	− 0.10 to 0.27	n.s.
TBW, L	18	9.9	1.8	10.6	1.9	0.7	0.4	0.5 to 0.9	< 0.001
FFM, kg	18	12.9	2.4	13.7	2.5	0.9	0.5	0.6 to 1.1	< 0.001
FFM, %	18	77.7	5.1	80.5	5.4	2.8	4.3	0.6 to 4.9	< 0.05
FM, kg	18	3.8	1.5	3.4	1.4	− 0.4	0.8	− 0.84	n.s.
FM, %	18	22.3	5.1	19.5	5.4	− 2.8	4.3	− 4.9 to − 0.6	< 0.05

*GH* growth hormone, *BMI* body mass index, *TBW* total body water, *FFM* fat free mass, *FM* fat mass, *n.s*. not significant, *delta* value of parameter after 6 weeks of GH treatment minus value of parameter before GH treatment

### Energy expenditure

Energy expenditure before and 6 weeks after GH treatment is given in Table 3. After 6 weeks of GH treatment there was a significant mean increase of 5% in BMR [mean increase 0.18 ± 0.23 MJ/day (43 ± 55 kcal/day); 95% CI 0.03–0.32 MJ/day (7–76 kcal/day); *p* < 0.05; *n* = 12]. There was no significant difference between girls and boys.

**Table 3:** Energy expenditure before and after 6 weeks of GH treatment

	*n*	Before GH treatment		After 6 weeks GH treatment		Delta			*p* value
		Mean	SD	Mean	SD	Mean	SD	95% CI	
BMR, MJ/day (kcal/day)	12	3.65 (872)	0.51 (122)	3.82 (912)	0.57 (136)	0.18 (43)	0.23 (55)	0.03 to 0.32 (7 to 76)	< 0.05
TEE, MJ/day (kcal/day)	18	5.17 (1235)	0.98 (234)	5.51 (1316)	1.02 (244)	0.33 (79)	0.52 (124)	0.07 to 0.59 (17 to 141)	< 0.05
AEE, MJ/day (kcal/day)	12	0.87 (208)	0.45 (107)	1.07 (256)	0.66 (158)	0.19 (45)	0.62 (148)	− 0.20 to 0.59 (− 48 to 141)	n.s.
PAL	12	1.37	1.13	1.43	0.20	0.05	0.19	− 0.07 to 0.17	n.s.
Physical activity, megacounts/day	12	2706	531	2503	457	− 265,240	563,400	− 736,254 to 205,775	n.s.

*GH* growth hormone, *BMR* basal metabolic rate, *TEE* total energy expenditure, *AEE* activity energy expenditure, *PAL* physical activity level, *n.s*. not significant, *delta* value of parameter after 6 weeks of GH treatment minus value of parameter before GH treatment

TEE also increased significantly (7%) after 6 weeks of GH treatment [mean increase 0.33 ± 0.52 MJ/day (79 ± 124 kcal/day); 95% CI 0.07–0.59 MJ/day (17–141 kcal/day); *p* < 0.05].

The increase in BMR was not significantly different from the increase in TEE [difference = 0.24 ± 0.67 MJ/day (57 ± 160 kcal/day); *p* = 0.249; *n* = 12].

Figure 1 illustrates the changes in BMR and TEE for each individual subject.

The BMR, estimated by the Oxford formula (Henry 2005) was not significantly different from the observed BMR measured by the ventilated hood method before start of GH treatment.

There was no significant increase in AEE, PAL and Tracmor counts per day.

The mean respiratory quotient (RQ) before GH treatment was 0.82; 6 weeks after GH treatment 0.84. This was not significantly different.

### Energy expenditure in relation to body composition

BMR was strongly related to FFM before start of GH treatment (*r* = 0.92, *R*^2^ = 0.84, linear equation: *y* = 1.21 + 0.2 × *x*). After 6 weeks of GH treatment this relation was similar (*r* = 0.76, *R*^2^ = 0.58, linear equation: *y* = 1.49 + 0.18 × *x*).

The change in TBW, FFM and FM was not related to the change in BMR and TEE (*r* ≤ 0.1, *R*^2^ ≤ 0.01).

### Energy expenditure in relation to first-year growth response

After the first year of GH treatment mean height was − 2.15 SD. The mean increase in height (∆Ht) SDS was 0.73 SD. Fourteen out of 18 patients (78%) had a good growth response (∆Ht SDS > 0.5).

**Fig. 2:**
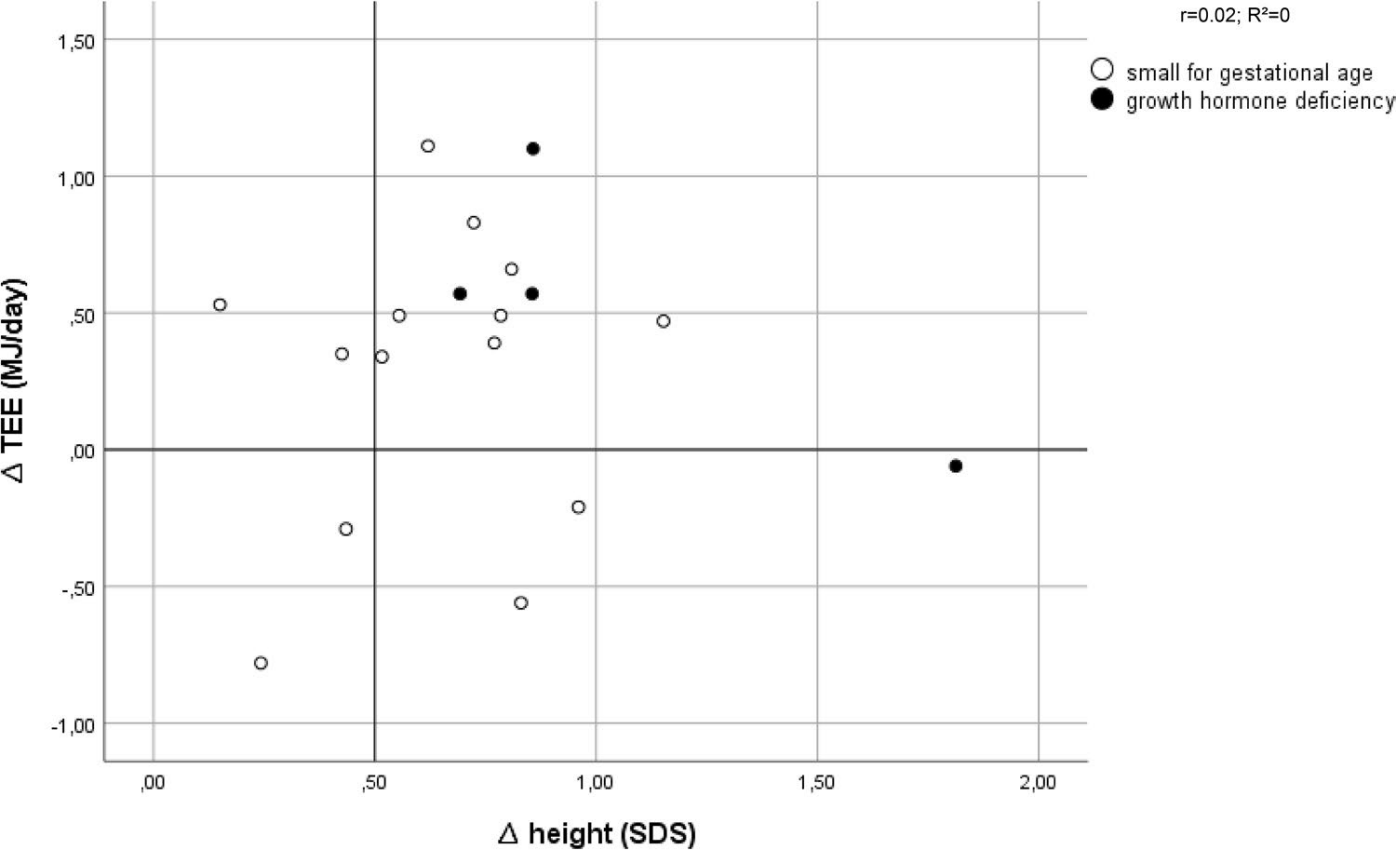
Effect of growth hormone treatment on total energy expenditure (TEE) in relation to first-year changes in height SDS (∆Ht SDS)

**Fig. 3:**
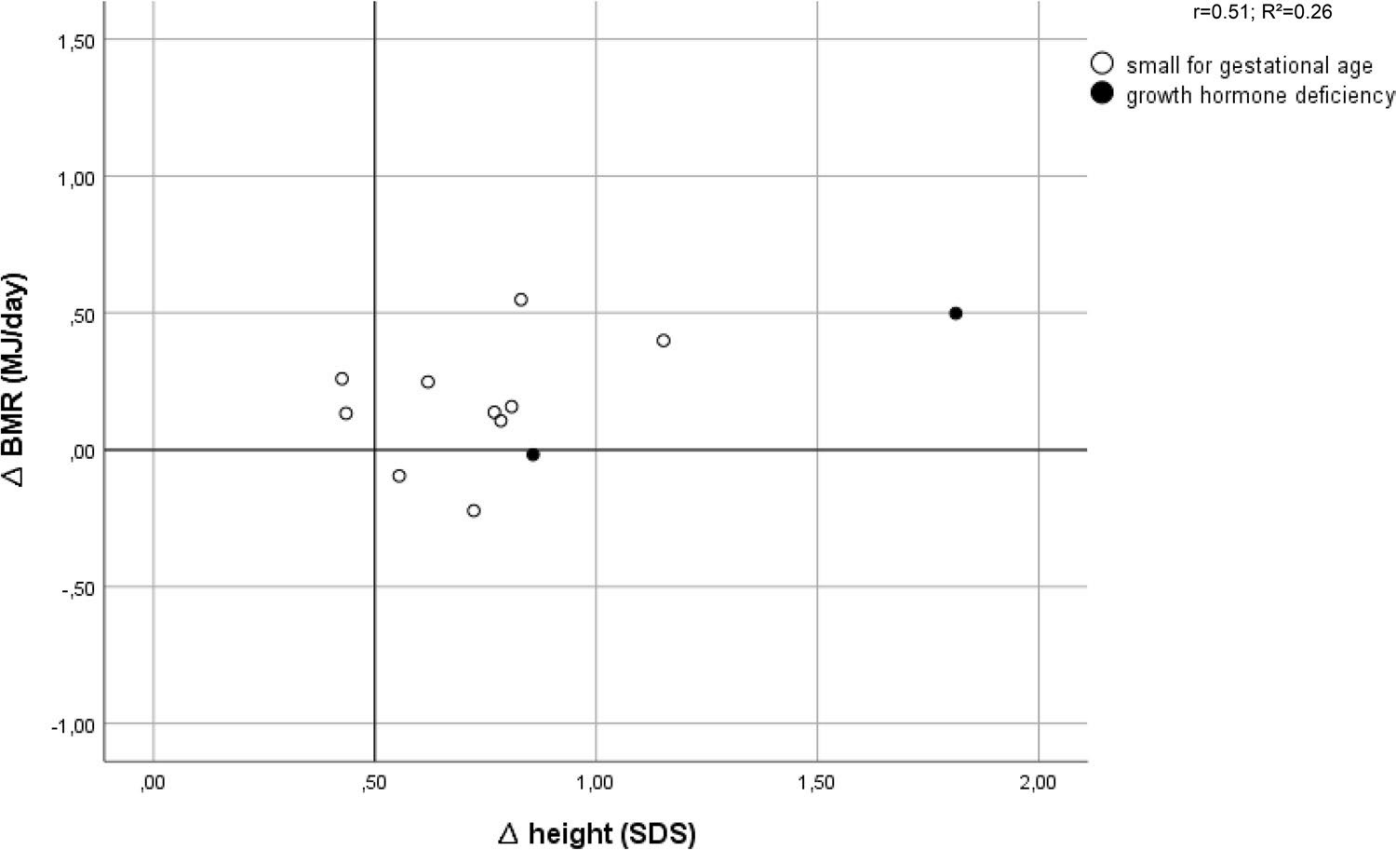
Effect of growth hormone treatment on basal metabolic rate (BMR) in relation to first-year changes in height SDS (∆Ht SDS)

**Fig. 4:**
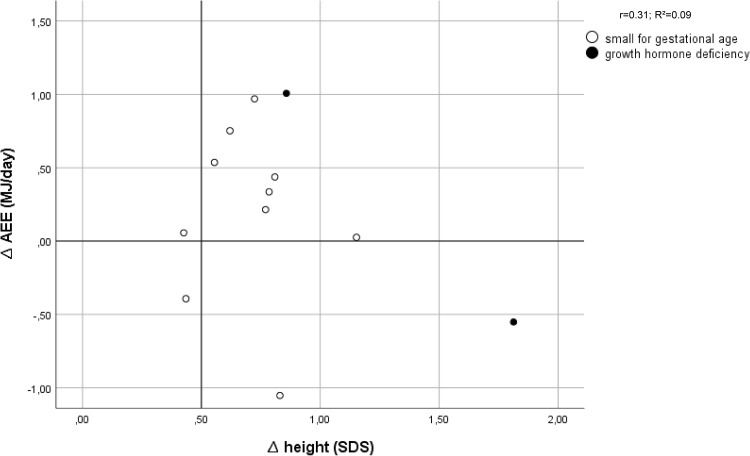
Effect of growth hormone treatment on activity related energy expenditure (AEE) in relation to first-year changes in height SDS (∆Ht SDS)

Thirteen out of 18 patients had an increased TEE after 6 weeks of GH treatment. Figure 2 shows that 11 out of these 13 patients (85%) had a good growth response after 1 year of GH treatment. For BMR and AEE this was 7 out of 9 patients and 8 out of 9 patients, respectively (*n* = 12) (Figs. 3, 4).

The children with no increase in TEE had varying growth responses and the few patients with poor growth response (4/18) showed varying responses in TEE.

GH dose was not related to ∆TEE (*r* = 0.19, *R*^2^ = 0.04) and ∆height SDS (*r* = 0.12, *R*^2^ = 0.02).

## Discussion

This study shows that 6 weeks of GH treatment has a positive effect on energy expenditure and body composition in children. Body composition changed by an increase in FFM as was demonstrated before in several studies (Ernst et al. 2012; Vaisman et al. 1992, 1994; Gregory et al. 1991, 1993; Walker et al. 1990; Khadilkar et al. 2014; Boot et al. 1997; Hassan et al. 1996). At the same time, total energy expenditure, measured by the DLW technique and energy expenditure at rest, measured by the ventilated hood method, showed an increase by 7% and 5% respectively. These results are comparable with the few other studies performed. Vaisman et al. (1994) showed a 13% increase in BMR after 2 months of GH treatment in 10 prepubertal boys with subnormal spontaneous GH secretion, and remained stable thereafter. Gregory et al. (1991) demonstrated a significant increase in BMR (12%) and TEE (7%) after only 6 weeks of GH treatment in 15 children (GHD, idiopathic short stature, Turner syndrome).

No relation between ∆FFM or ∆FM and ∆BMR or ∆TEE was observed. This is probably due to the relatively small cohort size and the dispersion of the data. Another explanation might be the relatively long observation period of 6 weeks, since the anabolic effect of GH, indicated by nitrogen retention increases within 24 h and reaches a maximum less than 2 weeks after initiation of treatment, followed by a gradual return of nitrogen excretion toward control levels after 2–3 weeks (Henneman and Henneman 1960). Gregory et al. (1991) found that the increase in BMR was significantly associated only with fat mass and not with fat free mass.

The RQ did not significantly change during GH treatment. However, based on the knowledge that GH increases lipid oxidation and decreases glucose oxidation, and based on the few available literature a decrease of the RQ would have been expected. Acute suppression of RQ during GH infusion has been reported (Jorgensen et al. 1993; Moller et al. 1990) and an increase in RQ following successful transsphenoidal surgery in acromegalic patients has been described (Moller et al. 1992b). Additionally, a more prolonged subcutaneous GH administration caused a decreased RQ in adults (Jorgensen et al. 1994; Moller et al. 1992a). To our knowledge, only one report described the effect of subcutaneously administered GH on RQ in children (Carrel et al. 1999). In 35 children with Prader Willi syndrome the RQ decreased after 12 months of GH treatment. We have no clear explanation why the RQ in our cohort did not decrease after 6 weeks of GH treatment.

Hoos et al. (2004) found that children who respond well to GH therapy (∆Ht SDS > 0.7) showed increased physical activity after 2 weeks of therapy as assessed with a tri-axial accelerometer. In contrast, in our study we observed no increase in PAL, Tracmor counts/day, nor in AEE after 6 weeks of GH treatment. Gregory et al. (1991) also concluded that GH has no discernible effect on activity levels. Therefore, it is reasonable to assume that GH has no effect on activity levels in children and that the increased energy expenditure is mainly used to increase metabolism in favour of growth.

We observed that 11 out of 13 children with an increased TEE had a good first-year growth response. On the other hand, good and poor first-year growth responders were indistinguishable from each other when TEE did not increase. Based on these results, the increase in TEE is not a tool to detect poor growth responders, but is very predictive for a good first-year growth response to GH treatment (∆Ht SDS > 0.5).

GH dose could be a possible cause for the differences in growth response, since it has been proven that GH dose affects height velocity during the first treatment year (Ranke 2003). However, GH dose does not explain the variations in first-year growth and ∆TEE in our cohort because our patients received the same dose throughout the whole first treatment year (SGA 35 mcg/kg/day and GHD 25 mcg/kg/day, according to the guidelines), except for 2 patients. As far as we know, the patients were compliant to the GH treatment. Other parameters known to be predictive for first-year height velocity such as age and weight at start of GH treatment, midparental height SDS, and birth weight SDS (Ranke 2009) were not significantly different between our good and poor growth responders.

The actual cause-effect relationship between TEE and growth can not be proven from this data. However, the most prominent metabolic effect of GH is a marked increase in lipolysis with mobilization of large quantities of free fatty acids from the adipose tissue. In addition, in the tissues throughout the body GH enhances the conversion of these fatty acids to acetylcoenzyme A which is used to supply most of the energy for the body cells, thus acting as a potent “protein sparer”. Some research workers have considered the protein-sparing effect to be a major factor that promotes protein deposition and growth (Black et al. 1986). Therefore, it is plausible to assume that an increased TEE leads to growth.

In conclusion, GH treatment showed a positive effect on body composition and energy expenditure in prepubertal children after 6 weeks of treatment. Despite these positive changes we were not able to demonstrate a relation between the increases in both effects of GH. No effect on physical activity was observed. Increase in TEE appeared to be valuable for the prediction of good growth responders to GH treatment.

## Abbreviations

AEE: Activity related energy expenditure
BMR: Basal metabolic rate
Cnts/d: Counts per day
DIT: Diet-induced thermogenesis
DLW: Doubly labelled water
GH: Growth hormone
GHD: Growth hormone deficiency
HV: Height velocity
∆Ht: Increase in height
MPH: Midparental height
PAL: Physical activity level
RQ: Respiratory quotient
SD: Standard deviation
SDS: Standard deviation score
SGA: Small for gestational age
TBW: Total body water
TEE: Total energy expenditure
